# How do trait self-control constructs and discounting relate to each other and to modifiable risk factors for cardiovascular disease?

**DOI:** 10.1007/s10865-026-00640-y

**Published:** 2026-03-31

**Authors:** Lili L. Kókai, Diarmaid T. Ó. Ceallaigh, Anne I. Wijtzes, Joost Oude Groeniger, Kirsten I. M. Rohde, Hans van Kippersluis, Alex Burdorf

**Affiliations:** 1 Department of Public Health, Erasmus MC, Dr. Molewaterplein 40, 3015 GD Rotterdam, The Netherlands; 2 Division of Human Nutrition and Health, Wageningen University & Research, Stippeneng 4, 6708 WE Wageningen, The Netherlands; 3 Department of Economics, Maynooth University, Co. Kildare, Maynooth, Ireland; 4 Erasmus University Rotterdam, Burgemeester Oudlaan 50, 3062 PA Rotterdam, The Netherlands; 5 School of Business and Economics, Maastricht University, Tongersestraat 53, 6211 LM Maastricht, The Netherlands

**Keywords:** Trait self-control constructs, Delay discounting, Probability discounting, Behavioral risk, Cardio metabolic risk, Cardiovascular health

## Abstract

Purpose: This study examines how different trait self-control constructs and discounting relate to each other and to modifiable behavioral and cardiometabolic risk factors for cardiovascular disease (CVD). Data from 8453 adults (18–65 years) participating in the Lifelines Cohort and the LIFESTYLE Study were analyzed. Participants self-reported on trait self-control constructs (Brief Self-Control Scale Short-form, Grit Scale Short-form, Delaying Gratification Inventory Short-form, Abbreviated Impulsiveness Scale, Risk Propensity Scale), delay discounting (exponential discounting and present bias parameters of the quasi-hyperbolic discount model elicited using choice lists), probability discounting (risk premium elicited using a certainty equivalent task), physical activity, sedentary behavior, fat and sugar intake, and sleep duration. Body mass index, waist–hip ratio, and pulse pressure were measured during a Lifelines clinic visit. Results: Measures of trait self-control constructs showed moderate correlations with each other, and very weak correlations with discounting measures. Discounting measures correlated (very) weakly with each other. Regression analyses showed that the Brief Self-Control Scale Short-form, Grit Scale Short-form, and Delaying Gratification Inventory Short-form were significantly associated with all of studied outcomes in the expected direction—higher self-control linked to better health outcomes. Other trait self-control construct and discounting measures demonstrated weaker associations. Allowing measures to group into factors did not improve predictive power. Conclusion: Trait self-control constructs and discounting are very weakly to moderately related, suggesting they represent distinct facets of a broader construct. Certain trait self-control constructs are important predictors of CVD risk, highlighting potential targets for interventions aimed at improving cardiovascular health.

**Supplementary Information:**

The online version contains supplementary material available at 10.1007/s10865-026-00640-y.

## Introduction

It is a priority public health concern to decrease risk for a leading cause of mortality and disability worldwide: cardiovascular diseases (CVDs) (Kaptoge et al., 2019). The number of people with CVDs and associated costs are only expected to increase in the future (Khavjou et al., 2016; Levenson et al., 2002). Fortunately, a substantial proportion of CVD morbidity and mortality is preventable, as many risk factors of CVD are modifiable (WHO, 2007). Important behavioral risk factors for CVD are insufficient physical activity, sedentary behavior, high fat intake, high sugar intake, and insufficient or too much sleep (AHA, 2015; Celis-Morales et al., 2018; Malhotra & Loscalzo, 2009; WHO, 2007). Modifiable cardio metabolic risk factors for CVD, such as overweight and (abdominal) obesity and high blood pressure, often go hand in hand with behavioral risk factors for CVD (Cannon, 2007).

For most individuals, engaging in health-promoting behaviors necessitates considerable effort and cognitive resources (Eisenberg et al., 2019; Hofmann et al., 2012a). People need to override their default behavior, which has been frequently performed, is rewarding, and can be executed with little effort or thought, and replace it with an effortful, cognitively demanding behavior. Then, healthy behavior is highly contingent on an individual’s capacity to suppress unhealthy impulses (de Ridder & de Wit, 2006; Hagger, 2014). For example, for one to start eating healthy, they have to give up the highly rewarding (fatty-sugary) foods they habitually eat. Or for one to engage in practices that lead to longer sleep, they have to give up their usual and pleasant bedtime procrastination activities. Additionally, maintaining healthy behavior is dependent on peoples’ ability to sustain effortful behavior in the long-term (Duckworth & Gross, 2014). For example, the costs of physical activity are in the short run, while its benefits are uncertain and generally appear only in the long run. Then, to stick to regular exercise, it is necessary to keep myopic and risk averse cognitions and feelings in check.

Trait self-control and discounting (both delay and probability discounting) have been found to be important psychological predictors of behavioral risk factors for CVDs. Trait self-control and discounting influence behavior, from physical (in)activity, diet, to sleep, through mechanisms such as controlling thoughts, regulating emotions, inhibiting impulses, weighing uncertain future benefits versus certain present costs, and taking risks (Amlung et al., 2016; Anderson & Mellor, 2008; Andrade & Hoyle, 2023; Barsky et al., 1997; Chabris et al., 2008; Cobb-Clark et al., 2023; Dohmen et al., 2011; Friese et al., 2011; Guarana et al., 2021; Hagger & Hamilton, 2024; Hagger, 2013; Moffitt et al., 2011; Reimers et al., 2009; de Ridder et al., 2012). A recent meta-analysis of 351 effect sizes showed a modest positive relationship between trait self-control and physical activity, healthier eating, and healthier sleep, explaining 3–7% of the variance in the domains examined (Andrade & Hoyle, 2023).

Trait self-control is often defined as the capacity to overrule predominant urges and undesirable behaviors to support the attainment of long-term goals (Carver & Scheier, 2012; Metcalfe & Mischel, 1999). Trait—as opposed to state—self-control is thought to be relatively stable across situations and over time; individuals with high trait self-control are better at controlling their impulses than others (Gottfredson & Hirschi, 1990; Mischel et al., 1996). Yet, despite rich literature spanning decades, fundamental questions about the conceptualization of trait self-control remain unanswered (Wennerhold & Friese, 2023). There is an extraordinary diversity in the measures of trait self-control available (Friese et al., 2011; Hagger, 2013; Moffitt et al., 2011; de Ridder et al., 2012), with the validity and reliability of often-used measures generally considered high (Coutlee et al., 2014; Duckworth & Quinn, 2009; Hoerger et al., 2011; Meertens & Lion, 2008; Morean et al., 2014; Tangney et al., 2004).

The strength of correlations between different measures of trait self-control can vary greatly. Moreover, earlier studies included a limited number of measures, and typically focused on the difference between revealed preference measures (i.e., executive function and delay tasks, where a participant’s choices have tangible consequences for themselves) and stated preference measures (i.e., self- and other-report questionnaires, where there are no tangible consequences) rather than the differences within those categories (Duckworth & Kern, 2011; Duckworth & Schulze, 2009; Eisenberg et al., 2019; Saunders et al., 2018). As opposed to executive function and delay tasks, self-report measures of trait self-control are simple and quick to administer, which is a central reason why they are often used in studies. From self-report questionnaires, the Brief Self-Control Scale by Tangney and colleagues remains the most used trait self-control measure (Tangney et al., 2004). The literature’s reliance on this measure may be a barrier for identifying which (other) self-report measures of trait self-control are more predictive of specific CVD risk factors (Andrade & Hoyle, 2023). Trait self-control lacks coherence as a construct; data-driven ontologies are needed to provide a basis for a cumulative science of self-control (Eisenberg et al., 2019). Some argue that aggregating multiple measures of trait self-control may explain more variance in CVD risk than measures each do by themselves (Roberts et al., 2006). To not contribute to the confusion either at the conceptual or measurement levels of *trait self-control*, the current paper will use the broader term of *trait self-control constructs* here on to refer to these various related constructs.

Other important predictors of behavioral risk factors for CVD are delay and probability discounting (Amlung et al., 2016; Anderson & Mellor, 2008; Barsky et al., 1997; Chabris et al., 2008; Dohmen et al., 2011; Green et al., 2014; Reimers et al., 2009). Delay discounting is the tendency to devalue later utility flows relative to earlier flows (Ericson & Laibson, 2019). We define utility flows, a concept from economics, as the stream of costs and benefits that an individual incurs as a result of a choice. For example, the choice to exercise incurs immediate costs in terms of effort, discomfort and time and later benefits to health and wellbeing. Exponential delay discounting is where utility flows are devalued at a constant rate over time (Ramsey, 1928; Samuelson, 1937). Exponential discounting is generally considered as a feature of rational decision-making and doesn’t capture failures of self-control (Ericson & Laibson, 2019). Other models of delay discounting have been developed to explain self-control failures, most notably the hyperbolic and quasi-hyperbolic discounting models (Ainslie, 1975; Ainslie, 1992; Chung & Herrnstein, 1967; Herrnstein, 1961; Laibson, 1997; Mazur, 2013; Phelps & Pollak, 1968; Strotz, 1973). These models engender time inconsistency (i.e. failing to follow through on previous intentions), which are considered by economists to be manifestations of self-control failures. In the hyperbolic model, earlier delays (e.g. from now until one week from now) are discounted more steeply than later delays (e.g. from one week from now until two weeks from now). The quasi-hyperbolic, or present bias, model is a simplification of the hyperbolic model that emphasizes the differences in discounting between immediate and future outcomes–earlier delays are only discounted more steeply than later delays if the earlier delay is between now and a future timepoint. This steeper discounting is referred to as present bias. In this present bias model, delays between two future timepoints are discounted exponentially.

Economists theorize that present bias is the parameter that predicts self-control problems in the sense of failing to follow through on previous intentions, whereas exponential discounting is unrelated to such problems (Laibson, 1997). While exponential discounting makes an individual delay unpleasant tasks, without present bias they would carry out the task at the delayed point in time as planned and hence exhibit no gap between intention and behavior (i.e. time inconsistency). Indeed, the quasi-hyperbolic model is the most popular model of self-control failures in economics and much of the economics literature even proxies self-control exclusively by the time inconsistencies described by this model (Delaney & Lades, 2017; Ericson & Laibson, 2019).

Probability discounting describes the extent to which people over- or under-value utility flows as they become more uncertain (Green & Myerson, 2004). In particular, risk aversion has been well-documented, which describes how people prefer certain outcomes to probabilistic outcomes, even if both outcomes have the same expected value (Holt & Laury, 2002). Risk aversion may decrease the likelihood that an individual engages in risky health behaviors, due to the future health risks these behaviors entail (Anderson & Mellor, 2008). Indeed, a number of empirical studies have shown that risk aversion is negatively associated with risky health behaviors (Anderson & Mellor, 2008; Barsky et al., 1997; Dohmen et al., 2011). Note however that, at least theoretically, increased risk aversion can, under certain conditions, lead to less preventive health behavior (Dionne & Eeckhoudt, 1985; Jullien et al., 1999).

Existing empirical evidence examines only a small number of trait self-control construct and delay and probability discounting measures in relation to each other or to behavioral and cardio metabolic risk factors for CVD, and is limited by small sample sizes. Moreover, it is yet to be established how trait self-control construct measures relate to measures of delay and probability discounting. The relatively few studies that have previously examined the associations between trait self-control construct and discounting measures found that they relate to each other (very) weakly (Beauchaine et al., 2017; Becker et al., 2023; Herman & Stanton, 2022; König-Kersting & Trautmann, 2025; Schulz van Endert & Mohr, 2022). Why should trait self-control constructs be related to delay and probability discounting? Trait self-control, as defined previously, is the capacity to overrule predominant urges and undesirable behaviors to support the attainment of long-term goals. Given this emphasis on long-term goals, it seems plausible then that it might be associated with observable preferences that support long-term goal achievement, such as a preference for larger future rewards over smaller immediate rewards (lower delay discounting) and with an aversion to tempting but risky options (lower probability discounting). However, that is not to say that delay and probability discounting may be alternative measures of trait self-control. Rather, they can be seen as expressions of aspects of an individual’s capacity for self-control that can be observed from choices over time-delayed or probabilistic outcomes. Furthermore, they represent only a subset of the channels through which self-control may manifest. In particular, they do not capture avoidance behaviors, such as situation modification and habit formation, that research suggests those with high trait self-control rely on to avoid self-control conflicts (Galla & Duckworth, 2015; Hofmann et al., 2012b).

In sum, various self-report measures of trait self-control constructs and delay and probability discounting have been shown to predict behavioral risk factors for CVD. However, an examination of how various measures relate to each other and what their respective association with CVD risk is was limited in scope. There are three important unresolved empirical questions pertaining to these constructs. How do self-report measures of trait self-control constructs (1) relate to each other, (2) to delay and probability discounting, and (3) to various modifiable risk factors for CVD? This study aims to contribute to answering these questions by (i) assessing a large sample of adults in a population-based cohort, (ii) utilizing a wide range of measures of trait self-control constructs and delay and probability discounting, (iii) exploring the interrelationships between these measures, and (iv) examining whether these measures relate differentially to a wide range of behavioral and cardio metabolic risk factors for CVD.

## Materials and methods

### Study design

To investigate the research questions described above, we designed a three-wave longitudinal study, called the LIFESTYLE Study. This study was conducted among participants of the Lifelines Cohort. Lifelines is a prospective population-based cohort study examining in a unique three-generation design the health and health-related behaviors of ~ 170,000 persons living in the North of the Netherlands. It employs a broad range of investigative procedures in assessing the biomedical, socio-demographic, behavioral, physical, and psychological factors which contribute to the health and disease of the general population, with a special focus on multi-morbidity and complex genetics. Further detail on the cohort and recruitment is provided elsewhere (Scholtens et al., 2015; Sijtsma et al., 2021).

### Study population

In Lifelines data collection wave 1A (2007–2013) participants completed questionnaires on a wide range of topics including demographics, health, health behavior, and psychosocial aspects, and they visited the Lifelines clinic for the standardized collection of various objective health measurements. A random subsample of adults aged 18–65 who had consented to be contacted by email about add-on questionnaires, such as our LIFESTYLE study, was approached to participate in our LIFESTYLE study, which was an additional data collection on psychological factors related to health behavior (n = 55,500). Data collection for the current study consisted of two batches: some participants were invited to take part between October and November 2019 (n = 15,000), others between February and April 2021 (n = 40,500). The LIFESTYLE study was a three-wave longitudinal study with participants expected to complete three questionnaires: at baseline (t_0_), follow-up 1 (t_1_ = t_0_ + 1 week) and follow-up 2 (t_2_ = t_0_ + 5 weeks). Of the 55,500 (15,000 + 40,500) adults invited to participate in the current study, 10,169 completed the baseline questionnaire (18%). These 10,169 participants were invited to fill in the follow-up 1 questionnaire; 8453 of them completed the follow-up 1 questionnaire (83%). These 8453 participants were invited to fill in the follow-up 2 questionnaire; 6860 of them completed the follow-up 2 questionnaire (81%). The data analyzed in this paper comes from the sample of 8453 participants that completed LIFESTYLE follow-up 1, unless otherwise stated. Compared to the full population of Lifelines cohort participants in wave 1A (approx. 150,000), our sample of 8453 is slightly older, higher educated, more likely to be female and less likely to have been born outside the Netherlands (see supplementary material, Table A1). When comparing those within our sample of 8453 who have missing values for trait self-control construct variables to those without missing values, we find that those with missing values are younger, more likely to have a lower educational level and less likely to have a middle educational level (see supplementary material, Tables A2-4). Analyzing those with missing values for the discounting variables shows they are older, more likely to be female, more likely to have a lower educational level and less likely to have a higher educational level.

For analyses, we imputed missing values for relevant variables in our sample of 8453 participants using multiple imputation by chained equations. The imputation model included all variables used in the analyses (measures of trait self-control constructs and discounting, covariates, and behavioral and cardio metabolic risk factors for CVD). The imputations were run using regression equations (linear regressions for continuous variables and logistic regressions for categorical variables). Analyses were run separately within each imputed dataset and results were combined using pooled estimates following Rubin’s Rules (Rubin, 1987).

### Measures of trait self-control constructs

As described above, trait self-control lacks coherence as a construct (Eisenberg et al., 2019). To contribute to data-driven ontologies that can provide a basis for a cumulative science of trait self-control, we have selected to examine some of the most-used self-report measures of trait self-control constructs: the Brief Self Control Scale Short-form (Morean et al., 2014; Tangney et al., 2004), the Grit Scale Short-form (Duckworth & Quinn, 2009), the Delaying Gratification Inventory Short-form (Hoerger et al., 2011), the Abbreviated Impulsiveness Scale (Coutlee et al., 2014) and the Risk Propensity Scale (Meertens & Lion, 2008) in the follow-up 1 wave of the LIFESTYLE Study. The Brief Self Control Scale Short-form consists of two subscales: Self-discipline (3 items; Cronbach’s alpha (α) = .63), and Impulse control (4 items, α = .64); we used a composite sum score for analyses (α = .71). Likert scale answer options range from *1* = *Not at all like me* to *5* = *Very much like me* (Morean et al., 2014). The Grit Scale Short-form consists of the subscales of Consistency of interest (4 items, α = .75), and Persistence of effort (4 items, α = .68); we averaged the scores (summed scores and then divided by the number of items) for analyses (α = .76). Likert scale answer options range from *1* = *Not at all like me* to *5* = *Very much like me* (Duckworth & Quinn, 2009). The Delaying Gratification Inventory Short-form consists of one factor; we used a sum score for analyses (10 items, α = .63) (Hoerger et al., 2011). Likert scale answer options range from *1* = *Strongly disagree* to *5* = *Strongly agree*. The Abbreviated Impulsiveness Scale consists of three subscales: Attention (5 items, α = .69), Motor (4 items, α = .76), and Non-planning (4 items, α = .70); we used a composite sum score for analyses (α = .79). Likert scale answer options range from *1* = *Rarely/Never* to *4* = *Almost Always/Always* (Coutlee et al., 2014). The Risk Propensity Scale is a one factor instrument; we averaged the scores for analyses (7 items, α = .78). Likert scale answer options range from *1* = *Totally disagree* to *9* = *Totally agree* on items 1 to 6, and from *1* = *Risk avoider* to *9* = *Risk seeker* on item 7 (Meertens & Lion, 2008). For ease of interpretation the Abbreviated Impulsiveness Scale and the Risk Propensity Scale were reverse coded, so that for all measures of trait self-control constructs a higher score indicates higher self-control.

### Measures of delay and probability discounting

Delay discounting was measured using two standard monetary choice lists (Cohen et al., 2020) in the baseline wave of the LIFESTYLE Study. These choice lists were designed to measure the two delay discounting parameters of the quasi-hyperbolic model: the present bias parameter (beta) and the exponential discount factor (delta) (Laibson, 1997; O'Donoghue & Rabin, 1999; Phelps & Pollak, 1968). This type of measure has been shown to have predictive validity for health behaviors, academic performance and financial behaviors (Amlung et al., 2016; Chabris et al., 2008; Cohen et al., 2020; Reimers et al., 2009). In the first choice list, participants made hypothetical choices between receiving €160 in one week’s time and receiving €x immediately, with €x = €160 in the first choice and getting progressively smaller in subsequent choices. The second choice list was the same as the first list, except that the choices were between €160 in two weeks’ time and €x in one week. Using responses to these choice lists, we estimated present bias (beta) and the exponential discount factor (delta). The closer the value of the present bias parameter to 0 (in the range 0–1), the more present biased (i.e. the more the person underweights the value of delayed monetary rewards); a value of 1 indicates no present or future bias; and the higher the value (above 1), the more future biased (overweighing the value of delayed monetary rewards). The exponential discount factor, Delta, measures how an individual weighs a sooner (not necessarily immediate) monetary reward relative to a later monetary reward, and is a constant rate over time. In the case of a sooner reward that is immediate, delta tells us the additional weighting added to the immediate reward in addition to the overweighting added by present bias.

Probability discounting was measured using a standard certainty equivalence task (Hershey & Schoemaker, 1985) in the baseline wave of the LIFESTYLE study. This type of measure has been shown to have predictive validity for health and financial behaviors (Anderson & Mellor, 2008; Barsky et al., 1997; Cheung et al., 2022; Dohmen et al., 2011; Guiso & Paiella, 2008; Yang et al., 2022). Participants were asked what sure amount would make them indifferent between that sure amount and a 50% chance of winning €300 (note that this was elicited with one question, not with a choice list). This sure amount stated by the participant (i.e. their certainty equivalent) was used to calculate their normalized risk premium, which we used as our measure of probability discounting. A score of 0 indicates that the participant is risk neutral, a score above 0 that they are risk averse, and a score under 0 that they are risk seeking regarding monetary outcomes in a lottery setting. Note that a higher score on each of the discounting measures can be interpreted as indicative of higher self-control.

### Behavioral and cardio metabolic risk factors for CVD

Participants self-reported their behavioral risk factors for CVD in the follow-up 1 wave of the LIFESTYLE Study. Fat and sugar intake was measured with the Dietary Fat and Free Sugar Short Questionnaire (26 items, α = .60) asking about intake in the past week (Francis & Stevenson, 2013). Participants self-reported their hours of moderate to vigorous physical activity (all days of the week) and sedentary behavior (only weekdays) in the past week, as measured by the International Physical Activity Questionnaire (Craig et al., 2003), each of which associate with CVD risk independently (Vasankari et al., 2020). Participants also self-reported their sleep quantity (i.e., average hours of sleep per night in the past week).

BMI (kg/m^2^), waist and hip circumference (cm) (Koning et al., 2007), and systolic and diastolic blood pressure (mmHg) (Domanski et al., 2002) were measured in a standardized manner in the Lifelines clinic during Lifelines wave 2A (2014–2017). For the purpose of this study, data on waist and hip circumference were converted to waist-hip ratio (waist circumference divided by hip circumference), and systolic and diastolic blood pressure were converted to pulse pressure (systolic blood pressure minus diastolic blood pressure).

### Covariates

Age, gender (male, female), country of birth (Netherlands, other), educational level (lower [no primary school education to lower or preparatory secondary vocational education], middle [junior general secondary education to pre-university secondary education], higher [higher vocational education to university education]), and whether the participant was part of the first (2019; pre-COVID-19) or second (2021; post-COVID-19) batch of the LIFESTYLE study were considered covariates in our regression analyses. Data on these variables was self-reported during Lifelines wave 1A, with the exception of batch number, which was automatically generated by the online data capture tool.

### Statistical analysis

Descriptive statistics were used to characterize the study population. For all analyses except descriptives, measures of trait self-control constructs and discounting were standardized. Associations between measures of trait self-control constructs and discounting were examined using a two-tailed Pearson correlation matrix. *r* < 0.20 indicates a very weak, 0.20–0.39 a weak, 0.40–0.59 a moderate, 0.60–0.79 a strong, and *r* ≥ 0.80 a very strong correlation (Evans, 1996).

Factor analyses were performed using the Principal Axis Factoring extraction method to assess how the measures group together. We have done so to examine the extent to which those factors are interpretable, and if they can more strongly predict CVD risk factors than individual measures of trait self-control constructs and discounting. The number of factors was not prespecified. Promax rotations were used to allow for correlations between factors and to reduce cross-loadings between factors. Factors with an eigenvalue above 1 were retained, and we used the minimum acceptability threshold of factors explaining 50% of variation jointly (Streiner, 1994).

Linear regression analyses were used to assess the associations of trait self-control construct and discounting measures with modifiable risk factors for CVD, adjusted for covariates (gender, age, country of birth, educational level, batch number). Each regression included only one of the trait self-control construct or discounting measures, along with the covariates. Partial η^2^, the proportion of variance accounted for by the predictor variable, was reported; values between 0.01 and 0.059 indicate a small effect size, between 0.06 and 0.139 a medium effect size, and 0.14 or higher a large effect size (Richardson, 2011). Multicollinearity statistics were assessed: all variance inflation factor values were well within the acceptable range (Becker et al., 2014).

We saved factor scores for the retained factors from the factor analyses and carried out linear regressions of each CVD risk factor on these factor-score variables, adjusted for covariates. All analyses were conducted with IBM SPSS Statistics for Windows version 28.0 (IBM Corp., 2017). Significance levels of *p* < .05 and *p* < .01 were used to indicate significant correlations and associations. P-values were adjusted to correct for multiple testing using the False Discovery Rate method (Benjamini & Hochberg, 1995; Benjamini et al., 2006).

## Results

### Demographics

Tables 1 and 2 show the characteristics of the study population. Over half of participants (59%) were female. Over half of participants (60%) were between 40 and 59 years old. Nearly all participants were born in the Netherlands (98%). Most participants had a higher (40%) or a middle (37%) educational level. In 2024, the proportion of the general Dutch population aged 44 to 45 that had a higher educational level was 42%; 50% had a middle educational level (CBS, 2024). 2366 participants (28%) were part of the first batch of data collection, while 6086 (72%) were part of the second batch. On average, participants engaged in 11.5h of physical activity per week. In 2022, 50% of the general Dutch population has not reached the recommended 2.5h of physical activity per week (CBS, 2022). On average, participants were sedentary for 31h per 5 weekdays. Overall, the study population reported to be in the lower ranges of fat and sugar intake (Francis & Stevenson, 2013). Generally, participants slept about 8h per night, were slightly overweight (BMI 26), and had a somewhat higher than normal pulse pressure (55) (CDC, 2022; Dart & Kingwell, 2001). The average male (0.96) and female (0.86) participant had a waist-hip ratio that would classify them as having a substantially increased risk for metabolic complications (WHO, 2008).

**Table 1:** Descriptive statistics of the study population: demographics, trait self-control constructs and delay and probability discounting

Demographics		Total	n
Gender	Female Male	5021 (59%) 3432 (41%)	8453
Age	< 40 40–59 60 +	1607 (19%) 5109 (60%) 1737 (21%)	8453
Country of birth	Netherlands Other	8168 (98%) 160 (2%)	8328
Educational level	Lower Middle Higher	1892 (23%) 3069 (37%) 3285 (40%)	8246
Batch	Participated in 2019 Participated in 2021	2366 (28%) 6087 (72%)	8453
*Trait self-control constructs*			
Brief self-control scale short-form (range: 7–35)		24.25 (3.69)	8194
Grit scale short-form (range: 1–5)		3.65 (0.50)	8162
Delaying gratification inventory short-form (range 10–50)		35.32 (4.15)	8146
Abbreviated impulsiveness scale (range 13–52)		38.84 (4.86)	8178
Risk propensity scale (range 1–9)		6.45 (1.29)	8177
*Delay discounting measures*			
Present bias (continuous measure ≥ 0)		1.00 (0.12)	6465
Delta (exponential discount factor) (continuous measure ≥ 0)		0.95 (0.15)	6465
*Probability discounting measure*			
Risk premium (continuous measure)		−0.037 (0.558)	7908

Frequencies and percentages for categorical variables and means and standard deviation for continuous variables.

**Table 2:** Descriptive statistics of the study population: behavioral and cardio-metabolic risk factors for CVD

Behavioral risk factors	Total		n
Physical activity	Hours/week	11.52 (12.61)	7474
Sedentary behavior	Hours/5 weekdays	31.4 (15.42)	7433
Fat and sugar intake (range: 26–130)	Intake/week	44.31 (6.73)	7843
Sleep quantity	Hours/night	8.06 (1.04)	7729
Cardio-metabolic risk factors			
Body mass index	Kg/m^2^	25.87 (4.08)	8063
Waist-hip ratio	Waist/hip circumference in cm	0.90 (0.09)	8063
Pulse pressure	Systolic blood pressure–diastolic blood pressure in mmHg	54.89 (12.03)	8053

Means and standard deviations for continuous variables.

### Correlations between measures of trait self-control constructs and discounting

Table 3 shows the results of the correlation analysis. Measures of trait self-control constructs were moderately to weakly correlated with each other, and correlated very weakly with measures of discounting. Measures of discounting correlated (very) weakly with each other.

**Table 3:** Pearson correlations between measures of trait self-control constructs and delay and probability discounting (n = 8453)

	1	2	3	4	5	6	7
*Trait self-control construct measures*							
1. Brief self-control scale short-form	–						
2. Grit scale short-form	.55**						
3. Delaying gratification inventory short-form	.48**	.44**					
4. Abbreviated impulsiveness scale	.57**	.54**	.45**				
5. Risk propensity scale	.22**	.10**	.10**	.22**			
*Delay discounting measures*							
6. Present bias	.02	−.00	−.00	.03	.03*		
7. Delta (exponential discount factor)	.03*	.01	.05**	.03*	−.02	−.23**	
*Probability discounting measure*							
8*. *Risk premium	.01	.00	.05**	.01	-.02	.01	−.09**

*p < 0.05, **p < 0.01. Standardized scores. P-values adjusted to correct for multiple hypothesis testing across all tests shown in Tables 3, 6, 7 using the False Discovery Rate Method. All scoring recoded so that a higher score indicates higher self-control

### Factor analyses between measures of trait self-control constructs and discounting

Table 4 shows the results of the factor analyses. Three factors with an eigenvalue above 1 emerged, and explained 61% of variation together. Most measures of trait self-control constructs—the Brief Self-Control Scale Short-form, Grit Scale Short-from, Delaying Gratification Inventory Short-form, and the Abbreviated Impulsiveness Scale—loaded onto Factor 1. We label this factor *dispositional self-control*. The measures of delay discounting—present bias and delta—loaded onto Factor 2 in different directions, with delta loading more strongly. We label this factor *delay discounting*. A trait self-control construct measure (Risk Propensity Scale) loaded onto Factor 3. We label this factor *risk propensity*. As shown in Table 5, the factors correlated with each other very weakly to weakly.

**Table 4:** Eigenvalues, variance explained and factor loading from factor analyses of measures of trait self-control constructs and discounting (n = 8453)

Measure		Factor 1	Factor 2	Factor 3
		Dispositional self-control	Delay discounting	Risk propensity
	Eigenvalue	2.59	1.25	1.02
	Variance explained	32.34%	15.65%	12.78%
Brief self-control scale Short-form		.744	− .009	.076
Grit scale short-form		.759	− .020	− .100
Delaying gratification inventory short-form		.641	.014	− .068
Abbreviated impulsiveness scale		.705	− .009	.094
Risk propensity scale		− .015	− .001	.680
Present bias		.003	− .246	.050
Delta (exponential discount factor)		.009	.907	.009
Risk premium		.044	− .105	− .056

Standardized scores. Factor analyses were performed using the Principal Axis Factoring extraction method.

**Table 5:** Pearson correlation matrix of factors (n = 8453)

Component	Factor 1	Factor 2
	Dispositional self-control	Delay discounting
Factor 1 Dispositional self-control	–	.045
Factor 2 Delay discounting	.045	–
Factor 3 Risk propensity	.358	− .025

### Associations of measures and factors of trait self-control constructs and discounting with modifiable risk factors for CVD

Table 6 shows the results of linear regressions run to analyze the associations between modifiable risk factors for CVD (outcome variables) and measures of trait self-control constructs and discounting (explanatory variables). Given that all of the measures of trait self-control constructs and discounting were coded so that a higher value was indicative of higher self-control, we expected that any significant association between these measures and the outcomes of physical activity and sleep quantity would be positive, and that any significant associations with the other outcomes would be negative.

**Table 6:**
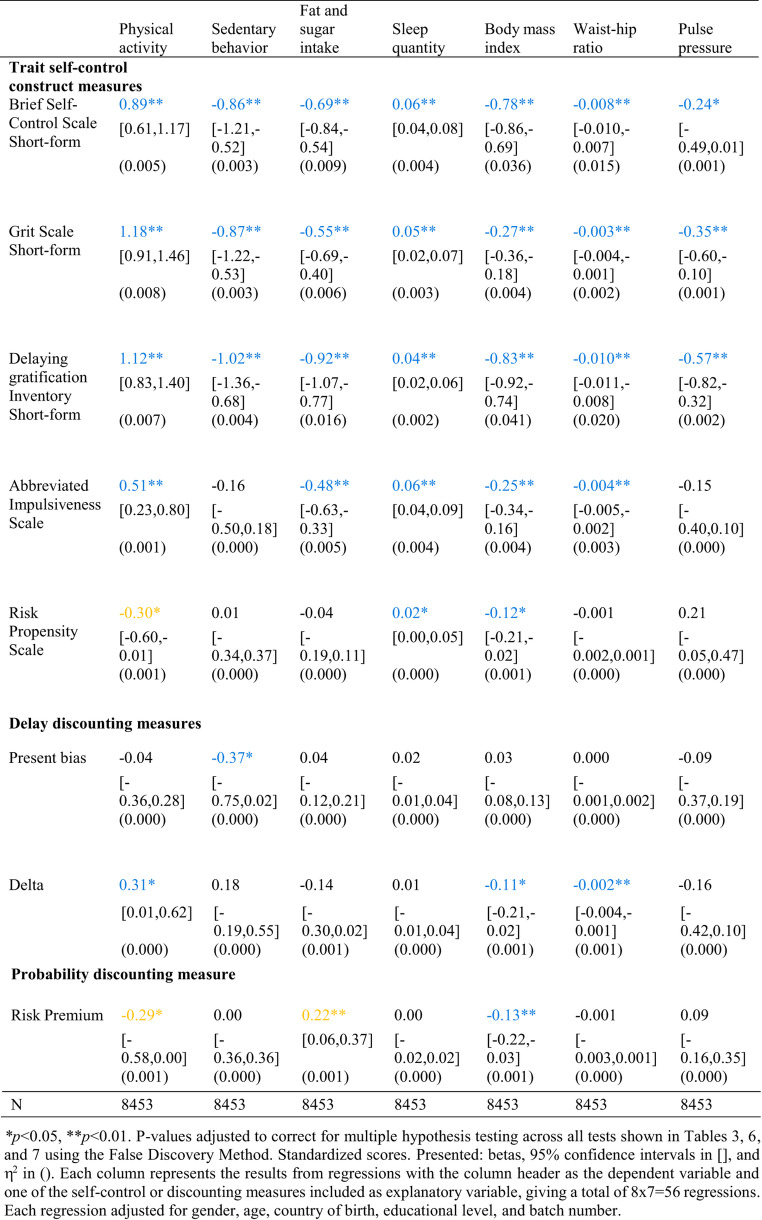
Regression coefficients, confidence intervals, and effect sizes for the associations of standardized measures of trait self-control constructs and discounting with modifiable risk factors for CVD

Significant associations in the expected direction are shown in blue in Table 6. The Delaying Gratification Inventory Short-form, the Brief Self-control Scale Short-form, and the Grit Scale Short-form were each significantly associated in the expected direction with all seven outcome variables. Most notably—considering clinical meaningfulness and effect size -, a one standard deviation (SD) increase in the Delaying Gratification Inventory Short-form and the Brief Self-Control Scale Short-form were associated with a 0.8 decrease in BMI; a one SD increase in the Grit Scale Short-form and the Delaying Gratification Inventory Short-form were associated with over 1h increase of physical activity per week; and a one SD increase in the Delaying Gratification Inventory Short-form, the Grit Scale Short-form, and the Brief Self-Control Scale Short-form were each associated with about 1h decrease of sedentary behavior per 5 weekdays. Next in performance was the Abbreviated Impulsiveness Scale (five significant associations in the expected direction), followed by Delta (three significant associations in the expected direction). All effect sizes, as measured by the partial η^2^, were (very) small.

A few significant associations in the opposite to the expected direction were also found. These are shown in orange in Table 6. Risk Premium showed two significant associations in the opposite direction to that expected, and the Risk Propensity Scale one. Independent associations between the CVD risk factors and trait self-control construct/discounting measures (i.e. associations for each measure when controlling for all other trait self-control construct/discounting measures) are shown in Table A5 in the supplementary material, and bivariate correlations between the CVD risk factors and the trait self-control construct/discounting measures are shown in supplementary material Table A7.

We checked for correlations between the CVD risk factors (supplementary material, Table A6). We find a small number of correlations greater than 0.2 in absolute terms, namely between physical activity and sedentary behavior (negative), and between BMI and each of waist-hip ratio and pulse pressure (positive). Given this, we additionally ran a MANCOVA analysis to test the relationship between each of the trait self-control and discounting measures and the combined set of CVD risk factors. This analysis showed a statistically significant relationship between the set of CVD risk factors and each of the trait self-control construct and discounting measures, except for present bias.

As shown in Table 7, only Factor 1, *dispositional self-control,* was significantly associated with some of the studied behavioral and cardiometabolic risk factors for CVD in a meaningful way. Most notably, a one-unit increase in *dispositional self-control* was associated with a 0.8 score decrease in BMI, a 1.7h increase of physical activity per week, and a 1.3h decrease of sedentary behavior per 5 weekdays. Again, all effect sizes were (very) small.

**Table 7:** Regression coefficients, confidence intervals, and effect sizes for the associations of factors of trait self-control construct and discounting measures with modifiable risk factors for CVD

	Physical activity	Sedentary behavior	Fat and sugar intake	Sleep quantity	Body mass index	Waist-hip ratio	Pulse pressure
Factor 1	1.68**	− 1.26**	−0.94**	0.08**	− 0.75**	− 0.009**	− 0.69**
*Dispositional* self-control	[1.33,2.02]	[− 1.69,− 0.83]	[− 1.13,− 0.76]	[0.05,0.11]	[− 0.87,− 0.64]	[− 0.011,− 0.007]	[− 1.01,− 0.37]
	(0.011)	(0.004)	(0.011)	(0.003)	(0.020)	(0.009)	(0.002)
Factor 2	0.04	0.43*	−0.14	0.005	− 0.10*	− 0.002**	− 0.11
*Delay discounting*	[− 0.25,0.33]	[0.07,0.79]	[− 0.29,0.02]	[− 0.02,0.03]	[− 0.19,− 0.003]	[− 0.004,− 0.001]	[− 0.37,0.16]
	(0.000)	(0.001)	(0.000)	(0.000)	(0.000)	(0.001)	(0.000)
Factor 3	− 1.26**	0.63*	0.31*	0.01	0.13	0.002	0.56**
*Risk propensity*	[- 1.71,- 0.81]	[0.08,1.18]	[0.07,0.55]	[- 0.03,0.04]	[− 0.02,0.27]	[− 0.001,0.004]	[0.15,0.96]
	(0.004)	(0.001)	(0.001)	(0.000)	(0.000)	(0.000)	(0.001)
N	8453	8453	8453	8453	8453	8453	8453

*p < 0.05, *^*^p < 0.01. P-values adjusted to correct for multiple hypothesis testing across all tests shown in Tables 3, 6, and 7 using the False Discovery Method. Standardized scores. Presented: betas, 95% confidence intervals in [], and η^2^ in (). Each column represents the results from a single regression with the column header as the dependent variable and with all three factors included as explanatory variables. Each regression adjusted for gender, age, country of birth, educational level, and batch number

### Robustness checks

In the three waves of the LIFESTYLE study, attention checks (“Please select the number 3 below”) were completed by participants. Excluding inattentive participants did not meaningfully alter the correlational, factor, or regression analyses results (available upon request).

In addition, in follow-up 1, participants were asked if they had a (medical) reason why they were not/less able to engage in physical activity or consume their regular diet in the studied period. Excluding participants that were not/less able to perform the behavior from the relevant regression analyses did not meaningfully alter the results (available upon request).

While we used hours of moderate to vigorous physical activity (MVPA) per week as our physical activity variable, the International Physical Activity Questionnaire also allowed us to calculate MET (metabolic equivalent of task) minutes per week (Craig et al., 2003). We ran the regressions on physical activity using this MET minutes per week variable and find no substantive difference in results compared to those obtained using the MVPA hours per week variable. There was a correlation of 0.9 between the two measures.

The Brief Self-Control Scale Short-form was administered to participants in each of the three waves of the survey to check whether this trait self-control construct is stable over time. We find the three measures to be strongly correlated (supplementary material, Tables A8–A10). We ran robustness checks using the average of the three Brief Self-control Scale Short-form measures—this did not substantively alter the correlational, factor, or regression analyses results (available upon request).

We ran all of our analyses using an available case sample without imputation, including only participants who had no missing values for any of the predictor or sociodemographic control variables we analyze (n = 5988). Results did not differ substantively from our main analyses with the imputed sample (supplementary material, Section B).

Finally we also ran the analyses using the K parameter from the hyperbolic discounting model as our measure of delay discounting (Mazur, 2013), instead of present bias and Delta which are derived from the *quasi-*hyperbolic discounting model. The hyperbolic discounting model is a precursor of the quasi-hyperbolic model, with the latter now being the more commonly used (Ainslie, 1975, 1992; Chung & Herrnstein, 1967; Herrnstein, 1961). This did not meaningfully alter the correlational, factor, or regression analyses results (available upon request).

## Discussion

This study set out to assess the relationship between different measures of trait self-control constructs and discounting, and their associations with key behavioral and cardio metabolic risk factors for CVDs.

In general, the self-reported measures of trait self-control constructs included in this study correlated moderately with each other. It is reassuring that these measures are correlated, as they were built to measure a common underlying trait. The finding that these correlations are not strong suggests that these measures of trait self-control constructs are not interchangeable, but likely measure different facets of a larger attribute. Moreover, these measures may also reflect domain-specificity: while none of these measures was explicitly designed to capture a trait self-control construct in specific domain (e.g. health), some may be better suited to capture one type of CVD risk factor (e.g. desirable, inhibition-related) over the other.

Measures of discounting correlated (very) weakly with each other and with measures of trait self-control constructs. The theoretical underpinning of the discounting measures shows that they capture different aspects of preferences that may, but need not, be correlated. The results of this study show that these aspects are only (very) weakly correlated. Present bias is typically thought to reflect self-control, especially among economists. Interestingly, this study finds only a very weak correlation between present bias and measures of trait self-control constructs, suggesting that present bias measures capture an aspect of behavior that is unrelated to the aspects of behavior that are captured by trait self-control construct measures. A three factor solution emerged from factor analysis, with measures of trait self-control constructs largely loading onto one factor (a factor we labelled *dispositional self-control*), measures of delay discounting loading onto another (labelled *delay discounting*), and the Risk Propensity Scale loading onto the final factor (labelled *risk propensity*). Taken together with the correlational results, the factor analysis results suggest that the measures of trait-self-control constructs included in this study may together measure a larger underlying attribute (Duckworth & Kern, 2011). The very weak correlation between the *dispositional self-control* and *delay discounting* factors is in line with the relatively few studies that have previously examined the associations between trait self-control construct and discounting measures (Beauchaine et al., 2017; Becker et al., 2023; Herman & Stanton, 2022; König-Kersting & Trautmann, 2025; Schulz van Endert & Mohr, 2022).

Regression analyses showed several significant associations in the expected direction. Of the studied measures of trait self-control constructs, the Brief Self-Control Scale Short-form, the Grit Scale Short-form, and the Delaying Gratification Inventory Short-form performed best in predicting CVD risk factors (physical activity, sedentary behavior, fat and sugar intake, sleep quantity, BMI, waist-hip ratio, pulse pressure): they related to all risk factors significantly and consistently in the expected direction (i.e. more self-control associated with better health outcomes). Our findings contradict some previous conclusions that the Brief Self-Control Scale would be more predictive of behaviors that are thought to be largely reliant on automatic processes (e.g. fat and sugar intake) than those that largely rely on deliberative processes (e.g. physical activity) (Andrade & Hoyle, 2023; Ridder et al., 2012). It should be noted that these studies used the 13-item full version of the Brief Self-Control Scale, instead of the 7-item short-form that the current study utilized. We have chosen to use the short-form to enhance data quality through its improved psychometric properties and reduced participant burden, as compared to the full version (Tangney et al., 2004). Our findings are in line with prior studies which identified the Grit Scale Short-form to be associated in a health-supportive manner with physical activity, sitting time and diet (Martin et al., 2023) and with exercise automaticity and weight loss maintenance (Gorin et al., 2024); and with those that found the Delaying Gratification Inventory to relate negatively to uncontrolled (i.e. episodes of loss of control or overeating) and emotional eating (e.g. subjective feelings of craving) (Cash & Breaux, 2025). Next in terms of performance was the Abbreviated Impulsiveness Scale, with five associations in the expected direction (physical activity, fat and sugar intake, sleep quantity, body mass index, waist-hip ratio). Again, our findings are in line with conclusions such as that impulsivity (as measured by the Barratt Impulsiveness Scale, the full version of the Abbreviated Impulsiveness Scale (Patton et al., 1995)) relates positively to uncontrolled eating (Lyke & Spinella, 2004). Delta showed three associations in the expected direction (physical activity, BMI, waist-hip ratio), but with small unit changes, comparable to previous findings (Anderson & Mellor, 2008; Bartels et al., 2023; Chabris et al., 2008). Present bias had only one significant association in the expected direction (sedentary behavior), despite being the most popular theoretical explanation for observed failures of self-control in economics.

We found three significant effects in the opposite direction of what we would expect. These were concentrated among the risk measures, namely the effects of the Risk Propensity Scale and Risk Premium on physical activity, and the effect of Risk Premium on fat and sugar intake. As noted in the introduction, theoretical economic research shows that increased risk aversion can, under certain conditions, lead to less preventive health behavior (Dionne & Eeckhoudt, 1985; Jullien et al., 1999). For example, someone who is highly risk averse may refrain from physical activity because they focus on the immediate risk of injury more than the future risk of CVD. Further research is needed to identify empirically the conditions under which risk aversion can lead to less preventive health behavior.

From the emerged factors, *dispositional self-control* showed associations of similar magnitude with outcome measures as individual measures of trait self-control constructs. *Delay discounting* and *risk propensity* showed little consistency in predicting risk factors, likely due to measures loading onto them in different directions and not predicting behavior strongly on their own to start with. Thus, the predictive power of measures was not increased by allowing them to group into factors. These findings challenge the idea that aggregating multiple measures of trait self-control constructs and discounting could explain more variance in CVD risk than they each do by themselves (Roberts, et al., 2006). Taken as a whole, our regression analyses on the individual measures and on factor scores show that measures of *dispositional self-control* are stronger predictors of CVD risk factors than *delay discounting* or *risk propensity*.

Effect sizes were small, generally explaining less than 1% of variation in risk factors, but this is not unexpected. Firstly, about half of physical activity and eating and a fifth of sleep-related behavior is governed by automatic, habitual processes, beyond the reach of self-control (Wood et al., 2002). Secondly, environmental factors also have an important influence on these risk factors (Sallis et al., 2016; Swinburn et al., 2011). Thirdly, prominent hypotheses describe that trait self-control acts by moderating the relationship between intention and health behavior (Hagger & Hamilton, 2024), and habit and health behavior (Phipps et al., 2025), rather than as a strong direct predictor. Finally, a meta-analysis of 351 effect sizes similarly found small positive relationships between trait self-control and physical activity, healthier eating, and healthier sleep, explaining 7%, 1% and 3%, respectively, of the variability in these outcomes (Andrade & Hoyle, 2023).

From a public health perspective, the identified unit changes could have a discernable contribution to reducing CVD risk at population level, providing support for a policy-level approach (Rose, 1981). On an individual level, it needs to be further examined what the practical implications of our findings are: is it feasible to strengthen (in the case of individuals high on trait self-control) or weaken (in the case of individuals low on trait self-control) the relationship between specific trait self-control constructs (e.g. delaying gratification) and CVD risk factors (e.g. physical activity) with an intervention program in order to achieve CVD health benefits? Due to a lack of intervention studies targeting the relationship between trait self-control constructs and CVD risk factors this remains an open question to be explored (Hagger, 2013, 2014). Alternatively, environmental adaptations need to me made such that self-control becomes less necessary for making healthy behavioral choices. By investing in interventions that create healthy situations and where the focus on people’s individual capacity to act is reduced, we could level the playing field of health behavior between those high and low on trait self-control (Duckworth et al., 2018).

### Limitations

Several limitations should be considered when interpreting the results of this study. Firstly, our study population,was different on some demographic variables than the full Lifelines Cohort from which our study population was drawn. Although mean differences on these variables were small between the groups, the generalizability of our results may be limited. Second, the cross-sectional nature of the data used means that causality cannot be inferred from our results, and that the temporal ordering between the assessed variables can only be assumed to a limited extent. However, as trait self-control and discounting are considered to be relatively stable across situations and time, and as one of our trait self-control construct measures proved to be stable over three time points, this may not compromise our results severely. Third, participating in a study requires deliberative thought, which means that self-reported data collected on impulsive processes, supposedly largely reliant on automatic cognitive processes, reflected individuals’ perceptions and experiences. Whether or not participants are aware of, or have access to, processes that are automatic and are purported to affect behavior beyond their awareness is an open question (Hagger et al., 2015). Empirical investigations find comparable validity between using self-reported versus physiological measures of automatic processes (most prominently, implicit association tests), with physiological measures showing less variability of effect size (Greenwald et al., 2009). Future studies should weigh the value of the added participant burden of using physiological measures, and choose measures accordingly in their study. Fourthly, the discounting measures involved the participant making numerical comparisons, and so measures may have carried some degree of noise, particularly for participants with lower numeracy. Such noise might have attenuated the strength of the associations we found between discounting measures and trait self-control construct measures and CVD risk factors. Fifthly, whereas we have adjusted for several covariates, we acknowledge there could be residual confounding where variables (e.g., cognition, family background) induce a spurious association between certain measures of trait self-control constructs, discounting and CVD risk factors. Finally, there were strong associations between some of our CVD risk factor outcome variables that were not accounted for in our analyses. Future studies could address this by carrying out a path analysis based on a theoretical model of how these variables might relate to each other.

## Conclusion

Taken together, our results show that measures of trait self-control constructs and discounting relate moderately to (very) weakly to each other, indicating that they capture different facets of a larger underlying attribute. Some of the measures of trait self-control constructs included in this study have shown to be important predictors of CVD risk. Interventions could aim to change the association between these constructs and risk factors for CVDs in a way that benefits the health of individuals, or create healthy environments that reduce the need for self-control.

## Conflict of interest

The authors have no relevant financial or non-financial interests to disclose.

## Consent to participate

Written informed consent was obtained from all participants included in the study.

## Consent for publication

Not applicable.

## Ethical approval

This study adheres most strictly to all applicable legal, ethical, and safety provisions of the Netherlands and the EU. The study was conducted in accordance with the principles of the Declaration of Helsinki. The Medical Ethics Review Board of the University Medical Center Groningen has approved the Lifelines Cohort (METc 2007/152), and the LIFESTYLE Study (METc-2019–464).

## Supplementary Information

Supplementary file 1.

## Data Availability

Data may be obtained from a third party and are not publicly available. Researchers can apply to use the Lifelines data used in this study. More information about how to request Lifelines data and the conditions of use can be found on their website (https://www.lifelines.nl/researcher/how-to-apply).
