# Supplementary material for: How do trait self-control constructs and discounting relate to each other and to modifiable risk factors for cardiovascular disease?
Source: J Behav Med. 2026 Mar 31;49(3):440–55. doi: 10.1007/s10865-026-00640-y (PMC13303321; doi:10.1007/s10865-026-00640-y)
Supplement: Supplementary file 1 [file 10865_2026_640_MOESM1_ESM.docx]

**Supplementary material**

**A. Additional tables**

**Table A1**

*Representativeness of study sample relative to full Lifelines cohort*

|  | Study sample | Lifelines Population | P-value for difference |
| --- | --- | --- | --- |
| Age (mean) | 49.0 | 44.3 | 0.000 |
| N | 8453 | 142785 |  |
|  |  |  |  |
| Gender |  |  |  |
| -Female (%) | 59.4 | 58.5 | 0.012 |
| -Male (%) | 40.6 | 41.5 |  |
| N | 8453 | 142888 |  |
|  |  |  |  |
| Education |  |  |  |
| -Lower (%) | 22.9 | 31.1 | 0.000 |
| -Middle (%) | 37.2 | 39.7 |  |
| -Higher (%) | 39.8 | 29.2 |  |
| N | 8246 | 141049 |  |
|  |  |  |  |
| Country of birth |  |  |  |
| -Netherlands (%) | 98.1 | 96.9 | 0.000 |
| -Other (%) | 1.9 | 3.1 |  |
| N | 8328 | 142761 |  |

Table compares the sociodemographic characteristics of our sample to that of the entire Lifelines cohort. For Age, p-value is from a t-test of equality of means. For the other variables, p-value obtained from a Pearson Chi-square test.

**Table A2**

*Difference in age between those with missing and non-missing values for each of the trait self-control construct and discounting measures*

|  | N | Mean | P-value for difference |
| --- | --- | --- | --- |
| Brief Self-Control Scale Short-form |  |  |  |
| -Non-missing | 8194 | 49.07 | 0.000 |
| -Missing | 259 | 45.41 |  |
|  |  |  |  |
| Grit Scale Short-form |  |  |  |
| -Non-missing | 8162 | 49.06 | 0.000 |
| -Missing | 291 | 45.93 |  |
|  |  |  |  |
| Delaying Gratification Inventory Short-form |  |  |  |
| -Non-missing | 8146 | 49.06 | 0.000 |
| -Missing | 307 | 46.04 |  |
|  |  |  |  |
| Abbreviated Impulsiveness Scale |  |  |  |
| -Non-missing | 8178 | 49.06 | 0.000 |
| -Missing | 275 | 45.81 |  |
|  |  |  |  |
| Risk Propensity Scale |  |  |  |
| -Non-missing | 8177 | 49.07 | 0.000 |
| -Missing | 276 | 45.66 |  |
|  |  |  |  |
| Present Bias |  |  |  |
| -Non-missing | 6465 | 48.18 | 0.000 |
| -Missing | 1988 | 51.48 |  |
|  |  |  |  |
| Risk Premium |  |  |  |
| -Non-missing | 7908 | 48.47 | 0.000 |
| -Missing | 545 | 55.96 |  |

Table compares the age of those in our sample for whom a given trait self-control construct/discounting variable is non-missing to the age of those for whom the variable is missing. P-values obtained from a t-test of equality of means. Comparison for the Delta discounting variable not shown as the missingness for this variable is identical to that for present bias (as both measures are obtained from the same choice list tasks).

**Table A3**

*Difference in gender between those with missing and non-missing values for each of the trait self-control construct and discounting measures*

|  | N | Female | Male | P-value for difference |
| --- | --- | --- | --- | --- |
| Brief Self-Control Scale Short-form |  |  |  |  |
| -Non-missing | 8194 | 59.5% | 40.5% | 0.534 |
| -Missing | 259 | 57.5% | 42.5% |  |
|  |  |  |  |  |
| Grit Scale Short-form |  |  |  |  |
| -Non-missing | 8162 | 59.5% | 40.5% | 0.405 |
| -Missing | 291 | 57.0% | 43.0% |  |
|  |  |  |  |  |
| Delaying Gratification Inventory Short-form |  |  |  |  |
| -Non-missing | 8146 | 59.5% | 40.5% | 0.384 |
| -Missing | 307 | 57.0% | 43.0% |  |
|  |  |  |  |  |
| Abbreviated Impulsiveness Scale |  |  |  |  |
| -Non-missing | 8178 | 59.5% | 40.5% | 0.587 |
| -Missing | 275 | 57.8% | 42.2% |  |
|  |  |  |  |  |
| Risk Propensity Scale |  |  |  |  |
| -Non-missing | 8177 | 59.4% | 40.6% | 0.623 |
| -Missing | 276 | 58.0% | 42.0% |  |
|  |  |  |  |  |
| Present Bias |  |  |  |  |
| -Non-missing | 6465 | 58.1% | 41.9% | 0.000 |
| -Missing | 1988 | 63.7% | 36.3% |  |
|  |  |  |  |  |
| Risk Premium |  |  |  |  |
| -Non-missing | 7908 | 59.0% | 41.0% | 0.011 |
| -Missing | 545 | 64.6% | 35.4% |  |

Table compares the gender of those in our sample for whom a given trait self-control construct/discounting variable is non-missing to the gender of those for whom the variable is missing. P-values obtained from a Pearson Chi-square test. Comparison for the Delta discounting variable not shown as the missingness for this variable is identical to that for Present Bias (as both measures are obtained from the same choice list tasks).

**Table A4**

*Difference in education between those with missing and non-missing values for each of the trait self-control construct and discounting measures*

|  | N | Low | Middle | High | P-value for difference |
| --- | --- | --- | --- | --- | --- |
| Brief Self-Control Scale Short-form |  |  |  |  |  |
| -Non-missing | 8001 | 22.7% | 37.4% | 39.8% | 0.014 |
| -Missing | 245 | 29.8% | 30.2% | 40.0% |  |
|  |  |  |  |  |  |
| Grit Scale Short-form |  |  |  |  |  |
| -Non-missing | 7971 | 22.7% | 37.5% | 39.8% | 0.011 |
| -Missing | 275 | 29.1% | 29.8% | 41.1% |  |
|  |  |  |  |  |  |
| Delaying Gratification Inventory Short-form |  |  |  |  |  |
| -Non-missing | 7956 | 22.8% | 37.5% | 39.8% | 0.026 |
| -Missing | 290 | 28.3% | 30.7% | 41.0% |  |
|  |  |  |  |  |  |
| Abbreviated Impulsiveness Scale |  |  |  |  |  |
| -Non-missing | 7986 | 22.7% | 37.5% | 39.9% | 0.004 |
| -Missing | 260 | 30.8% | 30.0% | 39.2% |  |
|  |  |  |  |  |  |
| Risk Propensity Scale |  |  |  |  |  |
| -Non-missing | 7985 | 22.7% | 37.5% | 39.8% | 0.006 |
| -Missing | 261 | 30.3% | 29.9% | 39.8% |  |
|  |  |  |  |  |  |
| Present Bias |  |  |  |  |  |
| -Non-missing | 6302 | 19.9% | 37.4% | 42.7% | 0.000 |
| -Missing | 1944 | 32.7% | 36.8% | 30.5% |  |
|  |  |  |  |  |  |
| Risk Premium |  |  |  |  |  |
| -Non-missing | 7716 | 21.2% | 37.7% | 41.1% | 0.000 |
| -Missing | 530 | 47.7% | 30.8% | 21.5% |  |

Table compares the education of those in our sample for whom a given trait self-control construct/discounting variable is non-missing to the education of those for whom the variable is missing. P-values obtained from a Pearson Chi-square test. Comparison for the Delta discounting variable not shown as the missingness for this variable is identical to that for present bias (as both measures are obtained from the same choice list tasks).

**Table A5**

*Independent associations of measures of trait self-control constructs and discounting with modifiable risk factors for CVD*

|  | Physical activity | Sedentary behavior | Fat and sugar intake | Sleep quantity | Body mass index | Waist-hip  ratio | Pulse pressure |
| --- | --- | --- | --- | --- | --- | --- | --- |
| **Trait-self control construct measures** | |  |  |  |  |  |  |
| Brief Self-Control Scale Short-form | 0.34 | -0.63* | -0.35** | 0.03* | -0.80** | -0.008** | 0.02 |
|  | [-0.03,0.72] | [-1.10,-0.15] | [-0.55,-0.14] | [0.00,0.06] | [-0.92,-0.68] | [-0.010,-0.006] | [-0.32,0.36] |
|  | (0.000) | (0.001) | (0.001) | (0.000) | (0.022) | (0.008) | (0.000) |
|  |  |  |  |  |  |  |  |
| Grit Scale Short-form | 0.92** | -0.66** | -0.07 | 0.01 | 0.29** | 0.004** | -0.21 |
|  | [0.57,1.28] | [-1.11,-0.21] | [-0.26,0.12] | [-0.02,0.04] | [0.18,0.40] | [0.002,0.006] | [-0.54,0.11] |
|  | (0.003) | (0.001) | (0.000) | (0.000) | (0.003) | (0.002) | (0.000) |
|  |  |  |  |  |  |  |  |
| Delaying gratification inventory Short-form | 0.78** | -0.84** | -0.77** | 0.00 | -0.72** | -0.009** | -0.57** |
|  | [0.44,1.12] | [-1.25,-0.43] | [-0.94,-0.59] | [-0.03,0.03] | [-0.83,-0.62] | [-0.011,-0.007] | [-0.87,-0.27] |
|  | (0.003) | (0.002) | (0.008) | (0.000) | (0.023) | (0.011) | (0.001) |
|  |  |  |  |  |  |  |  |
| Abbreviated Impulsiveness Scale | -0.40* | 0.90** | 0.06 | 0.04* | 0.36** | 0.002* | 0.14 |
|  | [-0.78,-0.02] | [0.44,1.36] | [-0.14,0.25] | [0.01,0.07] | [0.24,0.47] | [0.000,0.004] | [-0.20,0.48] |
|  | (0.000) | (0.002) | (0.000) | (0.001) | (0.005) | (0.001) | (0.000) |
|  |  |  |  |  |  |  |  |
| Risk propensity scale | -0.49** | 0.11 | 0.14 | 0.00 | 0.03 | 0.001 | 0.27* |
|  | [-0.80,-0.18] | [-0.27,0.48] | [-0.02,0.30] | [-0.02,0.03] | [-0.06,0.13] | [-0.001,0.003] | [-0.01,0.54] |
|  | (0.001) | (0.000) | (0.000) | (0.000) | (0.000) | (0.000) | (0.000) |
| **Delay Discounting measures** | | |  |  |  |  |  |
| Present bias | 0.04 | -0.35 | 0.03 | 0.02 | 0.02 | 0.000 | -0.14 |
|  | [-0.29,0.36] | [-0.74,0.04] | [-0.14,0.20] | [-0.01,0.04] | [-0.09,0.12] | [-0.002,0.002] | [-0.43,0.15] |
|  | (0.000) | (0.000) | (0.000) | (0.000) | (0.000) | (0.000) | (0.000) |
|  |  |  |  |  |  |  |  |
| Delta | 0.24 | 0.15 | -0.07 | 0.02 | -0.08 | -0.002* | -0.15 |
|  | [-0.07,0.55] | [-0.23,0.52] | [-0.23,0.09] | [-0.01,0.04] | [-0.18,0.02] | [-0.004,-0.000] | [-0.43,0.12] |
|  | (0.000) | (0.000) | (0.000) | (0.000) | (0.001) | (0.001) | (0.000) |
| **Probability Discounting measure** | | |  |  |  |  |  |
| Risk premium | -0.29* | 0.05 | 0.24** | 0.00 | -0.10* | -0.001 | 0.10 |
|  | [-0.58,-0.00] | [-0.31,0.41] | [0.09,0.39] | [-0.02,0.02] | [-0.19,-0.01] | [-0.002,0.001] | [-0.16,0.36] |
|  | (0.001) | (0.000) | (0.001) | (0.000) | (0.001) | (0.000) | (0.000) |
| N | 8453 | 8453 | 8453 | 8453 | 8453 | 8453 | 8453 |

**p*<0.05, ***p*<0.01. P-values adjusted to correct for multiple hypothesis testing using the False Discovery Method. Standardized scores. Presented: betas, 95% confidence intervals in [], and η^2^ in (). Each column represents the results from a single regression with the column header as the dependent variable and with all of the trait self-control construct and discounting measures included as explanatory variables. Each regression adjusted for gender, age, country of birth, educational level, batch number.

**Table A6**

*Pearson correlations between CVD risk factors and trait self-control construct and discounting measures* (n=8453)

|  | PA | Sed | FS | Sleep | BMI | WHR | PP |
| --- | --- | --- | --- | --- | --- | --- | --- |
| BSCS | .070^**^ | -.046^**^ | -.098^**^ | .062^**^ | -.192^**^ | -.068^**^ | -0.006 |
| Grit | .080^**^ | -.047^**^ | -.076^**^ | .044^**^ | -.076^**^ | -.043^**^ | -.043^**^ |
| DGIS | .066^**^ | -.041^**^ | -.128^**^ | .043^**^ | -.219^**^ | -.136^**^ | -.052^**^ |
| ABIS | 0.017 | 0.017 | -.049^**^ | .061^**^ | -.082^**^ | -.034^**^ | -.045^**^ |
| Risk prop. | 0.012 | -.039^**^ | -.045^**^ | .027^*^ | 0.005 | -.034^**^ | .090^**^ |
| PB | -0.004 | -0.025* | 0.006 | 0.014 | 0.007 | 0.002 | -0.010 |
| Delta | 0.020 | 0.023* | -0.019 | 0.019 | -.036^**^ | -.046^**^ | -0.010 |
| Risk prem. | -.035^**^ | 0.012 | .041^**^ | -0.002 | -.041^**^ | -0.001 | -0.007 |

**p*<0.05, ***p*<0.01. P-values adjusted to correct for multiple hypothesis testing using the False Discovery Rate Method.

**Table A7**

*Pearson correlations between CVD risk factors* (n=8453)

|  | *1* | *2* | *3* | *4* | *5* | *6* |
| --- | --- | --- | --- | --- | --- | --- |
| 1. Physical activity | - |  |  |  |  |  |
| 2. Sedentary behavior | -.269^**^ |  |  |  |  |  |
| 3. Fat and sugar intake | -0.012 | .114^**^ |  |  |  |  |
| 4. Sleep quantity | -.046^**^ | 0.018 | -.024^*^ |  |  |  |
| 5. Body mass index | 0.010 | 0.013 | -.078^**^ | -0.010 |  |  |
| 6. Waist-hip ratio | .065^**^ | -0.022* | 0.010 | 0.000 | .408^**^ |  |
| 7. Pulse pressure | .042^**^ | -0.011 | -.049^**^ | 0.012 | .214^**^ | .178^**^ |

**p*<0.05, ***p*<0.01. P-values adjusted to correct for multiple hypothesis using the False Discovery Rate Method.

**Table A.8**

*Repeating the Brief Self-Control Scale Short-form: means, standard deviations, n*

|  | *Mean* | *Standard deviation* | *n* |
| --- | --- | --- | --- |
| *Brief Self-Control Scale Short-form baseline* | 24.30 | 3.77 | 8445 |
| *Brief Self-Control Scale Short-form follow-up 1* | 24.25 | 3.69 | 8194 |
| *Brief Self-Control Scale Short-form follow-up 2* | 24.46 | 3.63 | 6884 |

**Table A.9**

*Correlations between Brief Self-Control Scale Short-form time points (n=8453)*

| Correlations | *Baseline* | *Follow-up 1* |
| --- | --- | --- |
| *Brief Self-Control Scale Short-form baseline* | - | - |
| *Brief Self-Control Scale Short-form follow-up 1* | 0.73** | - |
| *Brief Self-Control Scale Short-form follow-up 2* | 0.71** | 0.74** |

**Table A.10**

*Correlations between the average of the three Brief Self-Control Scale Short-form at three time points with the other trait self-control construct and discounting measures (listwise n=5988)*

|  | *1* | *2* |
| --- | --- | --- |
| *1. Brief Self-Control Scale Short-form x 3* | - |  |
| *2. Brief Self-Control Scale Short-form* | .95** |  |
| *3. Grit Scale Short-form* | .56** | .55** |
| *4. Delaying Gratification Inventory Short-form* | .49** | .48** |
| *5. Abbreviated Impulsiveness Scale* | .58** | .57** |
| *6. Risk Propensity Scale* | .23** | .22** |
| *7. Present Bias* | .03* | .02 |
| *8. Delta* | .03* | .03* |
| *9. Risk Premium* | .02 | .01 |

**p*<0.05, ***p*<0.01. Standardized scores. All scoring recoded so that a higher score indicates self-control. P-values adjusted for multiple testing using the False Discovery Rate method.

**B. Available case sensitivity analysis**

As a robustness check, we ran all of our analyses again using an available case sample without imputation, including only participants who had no missing values for any of the predictor or sociodemographic control variables we analyze (n=5988). The results of this analysis can be seen in Tables B1-B5 below. These results were consistent with the results from our main analysis carried out with the multiple imputation datasets. All point estimates were approximately equal, with no more than very minor and inconsequential differences, across the available case and multiple imputation analyses. Likewise for p-values. There were a very small number of differences in significance levels (i.e. significance stars), all occurring in cases where the p-value in both sets of analyses was very close to one of the threshold p-values (0.05 or 0.01), so that a small change in the p-value changed the significance level. Overall, there was no substantive difference between the results from the available case and multiple imputation analyses, and the conclusions that we draw from both sets of results are exactly the same.

**Table B.1**

*Pearson correlations between measures of trait self-control constructs and delay and probability discounting* (n=5988)

|  | *1* | *2* | *3* | *4* | *5* | *6* | *7* |
| --- | --- | --- | --- | --- | --- | --- | --- |
| Trait self-control measures |  |  |  |  |  |  |  |
| *1. Brief Self-Control Scale Short-form* | - |  |  |  |  |  |  |
| *2. Grit Scale Short-form* | .56** |  |  |  |  |  |  |
| *3. Delaying Gratification Inventory Short-form* | .49** | .44** |  |  |  |  |  |
| *4. Abbreviated Impulsiveness Scale* | .58** | .54** | .44** |  |  |  |  |
| *5. Risk Propensity Scale* | .23** | .11** | .10** | .24** |  |  |  |
| Delay discounting measures |  |  |  |  |  |  |  |
| *6. Present Bias* | .02 | .00 | .00 | .02* | .03* |  |  |
| *7. Delta (exponential discount factor)* | .03* | .01 | .05** | .02 | -.02 | -.24** |  |
| Probability discounting measure |  |  |  |  |  |  |  |
| *8. Risk Premium* | .01 | .00 | .04** | .00 | -.03* | .01 | -.10** |

**p*<0.05, ***p*<0.01. Standardized scores. P-values adjusted to correct for multiple hypothesis testing using the False Discovery Rate Method. All scoring recoded so that a higher score indicates higher self-control.

**Table B.2**

*Eigenvalues, variance explained and factor loading from factor analyses of measures of trait self-control constructs and discounting* (n=5988)

| **Measure** |  | **Factor 1**  *Dispositional self-control* | **Factor 2**  *Delay discounting* | **Factor 3**  *Risk aversion* |
| --- | --- | --- | --- | --- |
|  | **Eigenvalue** | 2.60 | 1.26 | 1.03 |
|  | **Variance explained** | 32.50% | 15.80% | 12.85% |
|  |  |  |  |  |
| *Brief Self-Control Scale Short-form* |  | .760 | -.004 | .070 |
| *Grit Scale Short-form* |  | .759 | -.023 | -.098 |
| *Delaying Gratification Inventory Short-form* |  | .641 | .014 | -.078 |
| *Abbreviated Impulsiveness Scale* |  | .701 | -.012 | .106 |
| *Risk Propensity Scale* |  | -.017 | .000 | .711 |
| *Present Bias* |  | .006 | -.244 | .041 |
| *Delta (exponential discount factor)* |  | .008 | .973 | .008 |
| *Risk Premium* |  | .042 | -.097 | -.061 |

Standardized scores. Factor analyses were performed using the Principal Axis Factoring extraction method.

**Table B.3**

*Pearson correlation matrix of factors* (n=5988)

| **Component** | **Factor 1**  *Dispositional self-control* | **Factor 2**  *Delay discounting* |
| --- | --- | --- |
| **Factor 1**  *Dispositional self-control* | - | .039 |
| **Factor 2**  *Delay discounting* | .039 | - |
| **Factor 3**  *Risk aversion* | .354 | -.037 |

**Table B.4**

*Regression coefficients, confidence intervals, and effect sizes for the associations of standardized measures of trait self-control constructs and discounting with modifiable risk factors for CVD*

|  | Physical activity | Sedentary behavior | Fat and sugar intake | Sleep quantity | Body mass index | Waist-hip  ratio | Pulse pressure |
| --- | --- | --- | --- | --- | --- | --- | --- |
| **Trait self-control construct measures** | | |  |  |  |  |  |
| Brief Self-Control Scale Short-form | 0.89** | -0.90** | -0.60** | 0.05** | -0.82** | -0.008** | -0.28* |
|  | [0.57,1.20] | [-1.30,-0.50] | [-0.77,-0.42] | [0.03,0.08] | [-0.92,-0.71] | [-0.01,-0.006] | [-0.57,0.01] |
|  | (0.005) | (0.003) | (0.008) | (0.003) | (0.041) | (0.013) | (0.001) |
|  |  |  |  |  |  |  |  |
| Grit Scale Short-form | 1.14** | -0.84** | -0.55** | 0.04** | -0.23** | -0.002* | -0.26 |
|  | [0.83,1.46] | [-1.24,-0.44] | [-0.72,-0.38] | [0.01,0.06] | [-0.33,-0.13] | [-0.004,0.000] | [-0.55,0.02] |
|  | (0.009) | (0.003) | (0.007) | (0.001) | (0.003) | (0.001) | (0.001) |
|  |  |  |  |  |  |  |  |
| Delaying gratification Inventory Short-form | 0.99** | -0.96** | -0.87** | 0.02 | -0.83** | -0.010** | -0.52** |
|  | [0.67,1.31] | [-1.36,-0.55] | [-1.04,-0.69] | [-0.004,0.05] | [-0.93,-0.72] | [-0.011,-0.008] | [-0.81,-0.23] |
|  | (0.007) | (0.004) | (0.017) | (0.000) | (0.042) | (0.019) | (0.002) |
|  |  |  |  |  |  |  |  |
| Abbreviated Impulsiveness Scale | 0.44* | -0.15 | -0.45** | 0.06** | -0.23** | -0.003** | -0.05 |
|  | [0.12,0.76] | [-0.55,0.26] | [-0.62,-0.28] | [0.04,0.09] | [-0.34,-0.13] | [-0.005,-0.001] | [-0.34,0.24] |
|  | (0.001) | (0.000) | (0.005) | (0.004) | (0.003) | (0.002) | (0.000) |
|  |  |  |  |  |  |  |  |
| Risk Propensity Scale | -0.47** | 0.07 | -0.02 | 0.04** | -0.10 | -0.000 | 0.40* |
|  | [-0.80,-0.14] | [-0.35,0.49] | [-0.20,0.16] | [0.01,0.07] | [-0.21,0.01] | [-0.002,0.002] | [0.10,0.70] |
|  | (0.001) | (0.000) | (0.000) | (0.001) | (0.001) | (0.000) | (0.001) |
|  | | |  |  |  |  |  |
| **Delay discounting measures** | | |  |  |  |  |  |
| Present bias | -0.02 | -0.41* | 0.06 | 0.01 | 0.01 | 0.000 | -0.11 |
|  | [-0.32,0.29] | [-0.80,-0.02] | [-0.11,0.23] | [-0.01,0.04] | [-0.09,0.11] | [-0.002,0.002] | [-0.40,0.17] |
|  | (0.000) | (0.001) | (0.000) | (0.000) | (0.000) | (0.000) | (0.000) |
|  |  |  |  |  |  |  |  |
| Delta | 0.36* | 0.27 | -0.09 | -0.01 | -0.15** | -0.003** | -0.19 |
|  | [0.04,0.67] | [-0.13,0.67] | [-0.26,0.08] | [-0.03,0.02] | [-0.25,-0.05] | [-0.005,-0.001] | [-0.48,0.09] |
|  | (0.001) | (0.000) | (0.000) | (0.000) | (0.001) | (0.002) | (0.000) |
| **Probability discounting measure** | | |  |  |  |  |  |
| Risk Premium | -0.52** | 0.09 | 0.22* | 0.00 | -0.15** | -0.001 | -0.02 |
|  | [-0.83,-0.20] | [-0.32,0.49] | [0.05,0.39] | [-0.02,0.03] | [-0.25,-0.04] | [-0.003,0.001] | [-0.31,0.27] |
|  | (0.002) | (0.000) | (0.001) | (0.000) | (0.001) | (0.000) | (0.000) |
| N | 5585 | 5543 | 5777 | 5715 | 5754 | 5754 | 5748 |

**p*<0.05, ***p*<0.01. P-values adjusted to correct for multiple hypothesis testing using the False Discovery Rate Method. Standardized scores. Presented: betas, 95% confidence intervals in [], and η^2^ in (). Each column represents the results from regressions with the column header as the dependent variable and one of the self-control or discounting measures included as explanatory variable, giving a total of 8x7=56 regressions. Each regression adjusted for gender, age, country of birth, educational level, and batch number.

**Table B.5**

*Regression coefficients, confidence intervals, and effect sizes for the associations of factors of trait self-control construct and discounting measures with modifiable risk factors for CVD*

|  | Physical activity | Sedentary behavior | Fat and sugar intake | Sleep quantity | Body mass index | Waist-hip  ratio | Pulse pressure |
| --- | --- | --- | --- | --- | --- | --- | --- |
| Factor 1  *Dispositional self-control* | 1.68** | -1.25** | -0.95** | 0.05** | -0.77** | -0.008** | -0.67** |
|  | [1.27,2.09] | [-1.77,-0.73] | [-1.17,-0.73] | [0.01,0.08] | [-0.90,-0.64] | [-0.011,-0.006] | [-1.04,-0.30] |
|  | (0.012) | (0.004) | (0.012) | (0.001) | (0.022) | (0.009) | (0.002) |
|  |  |  |  |  |  |  |  |
| Factor 2  *Delay discounting* | 0.26 | 0.36 | -0.04 | -0.01 | -0.11* | -0.002** | -0.14 |
|  | [-0.07,0.58] | [-0.05,0.77] | [-0.21,0.14] | [-0.03,0.02] | [-0.22,-0.01] | [-0.004,-0.001] | [-0.44,0.15] |
|  | (0.000) | (0.001) | (0.000) | (0.000) | (0.001) | (0.001) | (0.000) |
|  |  |  |  |  |  |  |  |
| Factor 3  *Risk aversion* | -1.35** | 0.70* | 0.38** | 0.04* | 0.16* | 0.003* | 0.84** |
|  | [-1.86,-0.85] | [0.06,1.35] | [0.10,0.65] | [0.00,0.09] | [-0.01,0.33] | [0.000,0.006] | [0.37,1.30] |
|  | (0.005) | (0.001) | (0.001) | (0.001) | (0.001) | (0.001) | (0.002) |
| N | 5585 | 5543 | 5777 | 5715 | 5754 | 5754 | 5748 |

*p<0.05, **p<0.01. P-values adjusted to correct for multiple hypothesis testing using the False Discovery Rate Method. Standardized scores. Presented: betas, 95% confidence intervals in [], and η^2^ in (). Each column represents the results from a single regression with the column header as the dependent variable and with all three factors included as explanatory variables. Each regression adjusted for gender, age, country of birth, educational level, and batch number.
